# Tris (2-chloroisopropyl) phosphate and Tris (nonylphenyl) phosphite Promote Human Renal Cell Apoptosis through the ERK/CEPBA/Long Non-Coding RNA Cytoskeleton Regulator Axis

**DOI:** 10.3390/toxics12070452

**Published:** 2024-06-22

**Authors:** Hu Zhao, Yi Yan, Yanyan Gao, Jiafu Wang, Sheng Li

**Affiliations:** Key Laboratory of Plant Resource Conservation and Germplasm Innovation in Mountainous Region (Ministry of Education), College of Life Sciences/Institute of Agro-Bioengineering, Guizhou University, Guiyang 550025, China; zhaohu8787@163.com (H.Z.); ezioauditore0624@gmail.com (Y.Y.); gaoyyian@163.com (Y.G.); jfwang@gzu.edu.cn (J.W.)

**Keywords:** organophosphate, lncRNA, apoptosis, CYTOR, CEBPA

## Abstract

Organophosphorus compounds (OPs) are widely used and have the potential to be harmful environmental toxicants to humans. Long non-coding RNA (lncRNA) plays a crucial regulatory role in cytotoxicity. This study aimed to investigate the effects of OPs on the expression of lncRNAs in cells. The effects of the industrial OPs TNPP and TCPP on both CYTOR and cellular viability were examined in the following human renal cell lines: HEK293T and HK-2. Both TCPP and TNPP downregulated CYTOR expression, increased reactive oxygen species levels, and induced apoptosis; the upregulated expression of CYTOR resulted in a reduction in apoptosis. The results of the luciferase reporter assay and the knock-down assay indicate that CEBPA binds to the upstream promoter region of CYTOR and regulates its transcription. Furthermore, TCPP and TNPP were found to downregulate the phosphorylation of ERK in the signaling pathway that is upstream of CEBPA. These results indicate that TCPP and TNPP can decrease the level of CEBPA by reducing ERK phosphorylation; this leads to a decrease in CYTOR expression, which further promotes cellular reactive oxygen species and apoptosis. Therefore, the ERK/CEBPA/CYTOR axis is one of the pathways by which organophosphates produce cytotoxicity, leading to renal cell injury. This study presents evidence for both the abnormal expression of lncRNA that is caused by organophosphates and the regulatory function of lncRNA regarding downstream cellular viability.

## 1. Introduction

Organophosphorus compounds (OPs) are widely used in the manufacture of agricultural pesticides, industrial flame retardants, and industrial antioxidants [1]. Since OPs do not degrade easily, they often enter the environment alongside agricultural products, construction materials, and commodity packaging—becoming environmental pollutants—and accumulate in the human body, causing toxicity to human organs and the nervous system [2]. TNPP are two organophosphorus compounds commonly used in industry. TCPP can be decomposed into carbon dioxide, phosphorus compounds, and hydrochloric acid to achieve flame retardancy in case of fire [3]; it is widely used in the production of rigid and flexible polyurethane foams [4]. The largest proportion of OPs found in airborne dust in some areas was reported to be TCPP, with a median value of 486 nmol/kg [5].On the other hand, TNPP is used as an antioxidant in the production of various plastics in order to retard or prevent polymer aging during processing and storage [6]. In a sample of indoor dust in one area, TNPP levels of 2763 nmol/g were reported [7]. In 2019, it was listed in the 21st batch of Substances of Very High Concern by the European Chemicals Agency. Therefore, it is important to further investigate the toxic effects that related organophosphorus compounds can have on humans and the environment.

As the main filtering and excretory organs, the kidneys play a crucial role in eliminating most toxic substances from the body in the form of prototypes or metabolites. Therefore, the kidneys are highly susceptible to damage from toxic substances [8]. Studies have shown that TNPP in food packaging bags can leach into the digestive tract, leading to nephrotoxicity and endocrine disruption [9]. At present, knowledge on the toxicological mechanisms of TCPP and TNPP is still incomplete and needs further study.

Long non-coding RNAs (lncRNAs) are a class of non-coding RNAs that are over 200 nt in length and are intricately linked to myriad pathological processes including autoimmune diseases and cancer [10,11]. A series of studies have shown that lncRNAs can interact when exposed to a variety of xenobiotics. For example, when the breast cancer cell MCF-7 was exposed to bisphenol A and diethyl sulfate, the transcription of lncRNA HOX antisense intergenic RNA (HOTAIR) was upregulated [12]. Additionally, studies have shown that lncRNAs play a role in the pathogenesis of smoking-induced Chronic Obstructive Pulmonary Disease [13,14]. Recent research has also shown that the lncRNA Gm10532 enhances m6A modification in chronic cadmium neurotoxicity, regulating FIS1-dependent mitochondrial fission [15]. Thus, lncRNAs are not only recognized as markers of environmental toxicant exposure, but also as key factors mediating the toxic effects of poisons. Cytoskeleton regulator (CYTOR) is a specific long non-coding RNA located on chromosome 2p11.2, and its abnormal expression is frequently linked to inflammation and cell apoptosis [16]. Our previous study found that the expression of some non-coding RNAs was downregulated in the human renal epithelial cell line HK-2 as a result of the stimulation of apoptosis via organophosphorus insecticide malathion [17]. However, how OPs affect CYTOR expression and exert toxic effects remains unclear.

The aim of this study was to resolve the toxic effects of the widely used OPs TCPP and TNPP on the human kidney cell lines HEK293T and HK-2. By elucidating the effects of these organophosphates on CYTOR expression and the regulatory role of CYTOR in the toxic effects, it will provide new insights into the mechanism of OP-induced lncRNA aberrant expression.

## 2. Materials and Methods

### 2.1. Cell Culture and Treatment

The human renal epithelial cell lines HEK293T and HK-2 were obtained from the China Center for Type Culture Collection (CCTCC, Wuhan, China); they were cultured and maintained in Dulbecco’s modified Eagle’s medium (DMEM) (Gibco, NY, USA) supplemented with 10% fetal bovine serum (FBS) (Gibco, USA), 1% penicillin, and 1% streptomycin (both from Solarbio) under 5% CO_2_ at 37 °C. TCPP (CAS No. 13674-84-5, Sigma-Aldrich, MO, USA) and TNPP (CAS No. 26523-78-4, Sigma-Aldrich, MO, USA) were dissolved in dimethyl sulfoxide (DMSO) to make stock solutions, which were then diluted with DMEM to different working concentrations. The control group cells were incubated with DMEM containing 0.1% DMSO.

### 2.2. Cell Viability Assay

Cells were seeded at a density of 1 × 10^3^ cells/mL per well in a 96-well plate; based on preliminary experiments, when the cell confluence reached 70–80%, the culture media containing different working concentrations of TCPP (0.05, 0.1, 0.25, 0.5, 1, and 2.5 mM) and TNPP (0.005, 0.01, 0.025, 0.05, 0.1, and 0.25 mM) were used for the treatment of HEK293T and HK-2 cells for 24 h, respectively. The experiments were performed five times independently for each treatment group. Then, the cell viability after the treatment was evaluated using the CCK-8 assay. Briefly, 20 μL of CCK-8 reagent (Beyotime Biotechnology, Shanghai, China) was added to each well. After 30 min of incubation, the OD values of the solution in each well at 450 nm were measured to calculate cell viability according to the following formula: cell viability (%) = (OD drug group − OD blank group)/(OD control group − OD blank group) × 100%. The IC_50_ values were calculated using GraphPad Prism 9.0 software based on the CCK-8 assay data.

### 2.3. Quantitative Real-Time PCR (qRT-PCR)

The total RNA from each group of cells was extracted using Trizol reagent (Servicebio, Wuhan, China). For lncRNA quantification, RNA was reverse-transcribed into cDNA using the lnRcute lncRNA cDNA synthesis kit (Cat No. KR202, TIANGEN Biotech. Inc., Beijing, China), and then qRT-PCR was performed using the cDNA and SYBR Green qPCR kit (Cat No. FP411, TIANGEN Biotech. Inc., Beijing, China). The lncRNA CYTOR primer sequences were as follows: 5′-ACCGAAAATCACGACTCAGCC-3′/5′-AATGGGAAACCGACCAGACC-3′. GAPDH was used as an endogenous reference and the primer sequences were as follows: 5′-GGAGTCCACTGGCGTCTTCA-3′/5′-GTCATGAGTCCTTCCACGATACC-3′. The CYTOR relative expression levels were calculated using the 2^−△△Ct^ formula.

### 2.4. Apoptosis Detection

Apoptotic cells were quantitated via flow cytometry by using propidium iodide (PI)/Annexin V conjugated (Annexin V-FITC) double staining (Keygen Biotech Co., Ltd., Nanjing, China). Briefly, after being treated with organic phosphorus for 24 h, the cells were washed with PBS three times, and were then stained under light-avoidance conditions according to the instructions of the Annexin V-FITC/PI cell apoptosis detection kit (Cat No. C1075S, Beyotime Biotechnology, Shanghai, China). After staining, the cells were analyzed using a flow cytometer FL2 detector (BD Biosciences, CA, USA), with an excitation wavelength set at 488 nm; FITC and PI signals were detected at emission wavelengths of 530 nm and 575 nm, respectively. Data analysis was performed using FlowJo 10.0.7 software. Each experiment was independently repeated three times.

### 2.5. Reactive Oxygen Species (ROS) Measurement

Cellular ROS levels were measured using the ROS Assay Kit (Cat No. MPC2203063, Servicebio, Wuhan, China). HK-2 and HEK293T cells were processed according to the manufacturer’s instructions. DCFH-DA fluorescence was excited at 488 nm and was detected at 525 nm using a flow cytometer. Data analysis was performed using FlowJo 10.0.7 software.

### 2.6. Transcription Factor Prediction

The transcription factors in the CYTOR gene promoter region (−2000 bp to +100 bp) were predicted using three transcription factor databases: PROMO, JASPAR, and Alibaba2.0. The intersection of the prediction results was used to screen for potential transcription factors and their binding sites for further testing, with a high level of credibility.

### 2.7. siRNA Interference Assay

The expression of transcription factors in HEK293T cells was inhibited using two independent small interfering RNAs (si-614 and si-1065). Cells were cultured in a six-well plate until the fusion rate reached approximately 30–50%; transfection experiments were performed using the transfection reagent Lipofectamine 3000 (Thermo Scientific, Waltham, MA, USA) according to the manufacturer’s instructions. At 6 h after transfection, the culture medium was refreshed. When cells had grown by approximately 80%, subsequent experiments were conducted after treatment with TNPP and TCPP, separately, for 24 h. The sequence of primers applied in siRNA interference were as follows:

si-614: 5′-GCGAGGAGGAUGAAGCCAATT-3′

si-614 anti-sense: 5′-UUGGCUUCAUCCUCCUCGCTT-3′

si-1065: 5′-GGAGCUGACCAGUGACAAUTT-3′

si-1065 anti-sense: 5′-AUUGUCACUGGUCAGCUCCTT-3′

si-NC: 5′-UUCUCCGAACGUGUCACGUTT-3′

si-NC anti-sense: 5′-ACGUGACACGUUCGGAGAATT-3′

### 2.8. Acetylcholinesterase (AChE) and Carboxylesterase (CES) Activity

The esterase activities of AChE and CES were determined using an enzyme-linked immunosorbent assay (ELISA). When the cells in the six-well plate were cultured to a confluence of about 70%, organophosphorus was treated for 24 h, the cells were collected via trypsin digestion, the cell suspension was quickly frozen with liquid nitrogen, and was then thawed in a 37 °C water bath 3 times to destroy the cells and release intracellular components. At 3000 rpm, the sample was centrifuged for 20 min, the supernatant was collected, and the sample to be tested was obtained. The activity was evaluated according to the instructions of the AChE, CES test kit (Cat No. TW27775, Tongwei Co., Ltd., Shanghai, China).

### 2.9. Dual Luciferase Assay

The CYTOR promoter luciferase-reporter plasmid was constructed by Tsingke Biotechnology Co, Ltd (Beijing, China). In brief, based on the prediction results from the database, a fragment of 200 bp was selected near the binding site of the transcription factor on the CYTOR gene promoter. This was divided into two groups—the wild-type group (CYTOR-WT) and the mutant group (CYTOR-MUT). Both were cloned into the pGL3-basic vector. The CDS sequence of the transcription factor CCAAT enhancer binding protein alpha (CEBPA) was constructed into the pcDNA3.1 vector. Subsequently, the two kinds of vectors were co-transfected into HEK293T cells that had been pre-cultured in a 6-well plate. The Renilla luciferase expressed by the internal reference plasmid (pRL-TK) was used to normalize for the transfection efficiency. The dual activities of firefly luciferase and Renilla luciferase were detected using the dual-luciferase reporter assay system (Promega, WI, USA).

### 2.10. Western Blot

After 24 h of treatment with different concentrations of OPs, the total protein of the cells was extracted using RIPA lysis buffer (Beyotime Biotechnology, Shanghai, China), and the concentration of each protein sample was determined and adjusted using a BCA protein quantification kit (Cat No. P0012S, Beyotime Biotechnology, Shanghai, China). Proteins of different sizes were separated using SDS-PAGE experiments, and then the proteins were transferred to PVDF membranes via wet transfer. After blocking with 5% skim milk for 1 h at room temperature, the primary antibody was incubated overnight at 4 °C, washed three times with TBST, incubated with secondary antibody for 1 h at room temperature, washed three times with TBST, and the target protein was developed with ECL (Beyotime Biotechnology, Shanghai, China) developing solution; the chemiluminescent imaging system was used to calculate the expression level. The primary antibodies used included phosph-p38 (Cat No. 28796-1-AP, 1:1000), p38 (Cat No. 14064-1-AP, 1:2000), phosph-ERK1/2 (Cat No. 80031-1-RR, 1:2000), ERK1/2 (Cat No. 11257-1-AP, 1:2000), CEBPA (Cat No. 29388-1-AP, 1:1000), GAPDH (Cat No. 80570-1-RR, 1:2000), and the secondary antibody Goat Anti-Rabbit IgG(H+L) (Cat No. SA00001-2, 1:10,000); all were from Proteintech Group, Inc (Wuhan, China).

### 2.11. Statistical Analysis

Experiments were repeated at least three times, and the data are expressed as mean ± SD (standard deviation). The experimental data were analyzed using SPSS 22.0 and Graphpad Prism 9.0. A two-tailed (*t*-test) was used to compare the differences between the two groups. One-way analysis of variance (ANOVA) and Fisher’s least significant difference (LSD) test were used to compare the differences between multiple groups. * indicates a *p* value < 0.05; ** indicates a *p* value < 0.01; and ns indicates not significant.

## 3. Results

### 3.1. TCPP and TNPP Inhibited the Viability of HEK293T and HK-2 by Inducing Apoptosis

**Figure 1:**
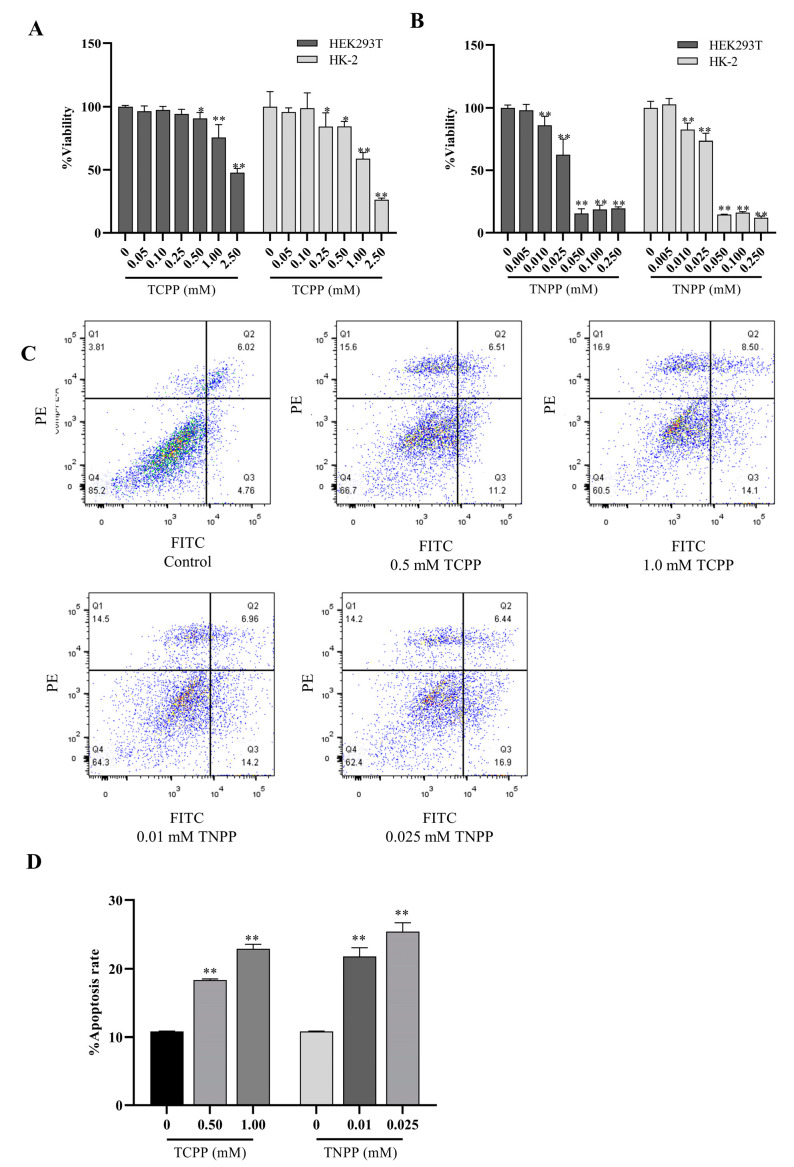
TCPP and TNPP inhibited the viability of HEK293T and HK-2 cells. (**A**) The viability of HEK293T and HK-2 treated with different concentrations of TCPP was determined using the CCK-8 assay. Data are presented as the mean ± SD (* *p* < 0.05, ** *p* < 0.01, *n* = 5). (**B**) The viability of HEK293T and HK-2 treated with different concentrations of TNPP was determined using the CCK-8 assay. Data are presented as the mean ± SD (** *p* < 0.01, *n* = 5). (**C**) Dot-plot graphs from flow cytometric analysis in HEK293T cells stained with Annexin V–FITC/PI dual stain after treatment with TCPP and TNPP, respectively. The colored dots represent cell density and the red dots represent the highest cell density. (**D**) Bar diagram depicting the apoptotic rates (%) in the indicated groups. Data are presented as the mean ± SD (** *p* < 0.01, *n* = 3).

The human renal cell lines HEK293T and HK-2 were exposed to six different concentrations of TCPP (0.05–2.5 mM) and TNPP (0.005–0.25 mM) for 24 h. The effects of TCPP and TNPP on the survival of both renal cell lines were evaluated using the CCK-8 assay. The results showed that both drugs had inhibitory effects on the viability of HEK293T and HK-2 cells at the concentrations tested (Figure 1A,B). The IC_50_ values of TCPP and TNPP were 1.392 mM and 0.0254 mM for HEK293T, and 1.099 mM and 0.0264 mM for HK-2, respectively. These results indicated that renal cells were more sensitive to TNPP than to TCPP. The forms of TCPP and TNPP that inhibit cell viability were examined using flow cytometry. As demonstrated in Figure 1C,D, both TCPP and TNPP caused cell apoptosis, and the rate of apoptosis increased with higher concentrations of treatment. These results suggest that causing apoptosis is an important method in which TCPP and TNPP inhibit renal cell viability.

### 3.2. TCPP and TNPP Downregulated CYTOR and Overexpressed the Apoptosis of CYTOR-Attenuated Cells

**Figure 2:**
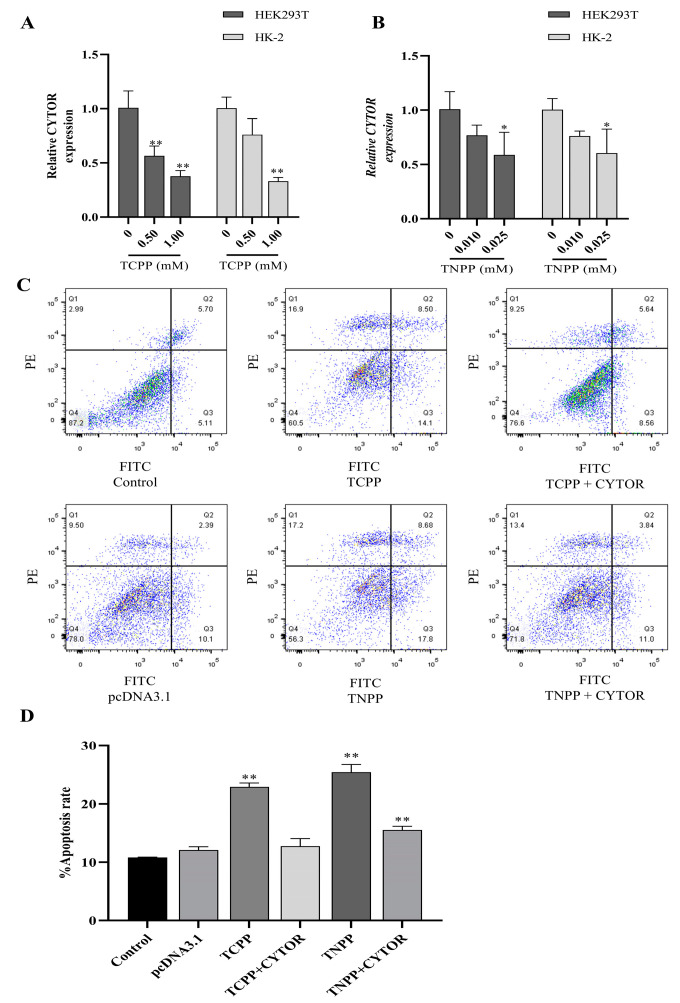
CYTOR reduces the induction of apoptosis by TCPP and TNPP. (**A**) The relative expression levels of CYTOR in HEK293T and HK-2 cells after TCPP treatment. Data are presented as the mean ± SD (** *p <* 0.01, *n* = 3). (**B**) The relative expression levels of CYTOR in HEK293T and HK-2 cells after TNPP treatment. Data are presented as the mean ± SD (* *p* < 0.05, *n* = 3). (**C**) Flow cytometric analysis of HEK293T cells treated with TCPP and TNPP overexpressing or not overexpressing CYTOR. The colored dots represent cell density and the red dots represent the highest cell density. (**D**) Bar diagram depicting the apoptotic rates (%) in the indicated groups. Data are presented as the mean ± SD (** *p* < 0.01, *n* = 3).

The qPCR assay results showed that TCPP (0.5 mM, 1.0 mM) and TNPP (0.01 mM, 0.025 mM) significantly downregulated the expression of CYTOR in both renal cells after treatment for 24 h in a dose-dependent manner (Figure 2A,B). After the transfection of HEK293T cells with the CYTOR overexpression vector, these cells were further exposed to TCPP and TNPP. As shown in Figure 2C,D, the apoptosis rate of cells in the transfected group was significantly reduced after TCPP and TNPP treatment compared with the untransfected group. These results suggest that the expression of CYTOR can attenuate renal cells apoptosis, which was induced by TCPP and TNPP.

### 3.3. TCPP and TNPP Increased the Cellular ROS Levels

**Figure 3:**
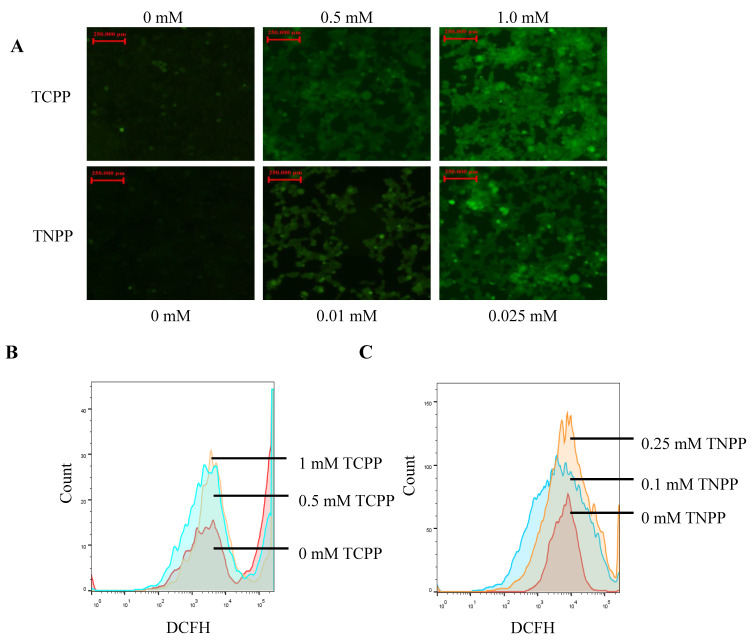
TCPP and TNPP increase ROS in HEK293T cells. (**A**) Detection of ROS levels in H293T cells treated with different concentrations of TCPP and TNPP using fluorescence microscopy with green fluorescent probe DCFH-DA. (**B**) Flow cytometry was used to detect the effects of 0.5 mM and 1 mM TCPP on the ROS content of HEK293T. (**C**) Flow cytometry was used to detect the effects of 0.25 mM and 0.1 mM TNPP on the ROS content of HEK293T.

The Dcfh-da probe was used to detect the effects of TCPP and TNPP on ROS levels in HEK293T cells. After treating the HEK293T cells with TCPP and TNPP for 24 h, the DCFH that entered the cells could be oxidized to DCF by ROS content, thus producing a denser and more intense green fluorescence (Figure 3A). The DCF signal was subsequently analyzed using flow cytometry, revealing a gradual increase in ROS content with increasing treatment concentration (Figure 3B,C). These findings indicate that low concentrations of TCPP and TNPP can decrease cell viability by increasing ROS levels.

### 3.4. TCPP and TNPP Had No Effect on Cellular Phosphotransferase Activity

**Figure 4:**
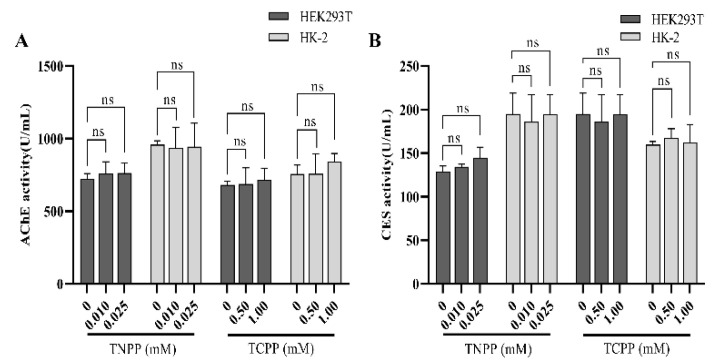
TNPP and TCPP did not affect the activities of intracellular AChE and CES at the assayed concentrations. (**A**) AChE activity in both kidney cells after TCPP vs. TNPP treatment. (**B**) CES activity in both kidney cells after TCPP vs. TNPP treatment. Data are presented as the mean ± SD (ns indicates not significant , *n* = 3).

AChE and CES are two enzymes in human cells that metabolize OPs. OPs can be neurotoxic by inhibiting the activity of AChE and CES after acute high-dose exposure [18,19]. The activities of AChE and CES were detected in HEK293T and HK-2 cells using the ELISA assay. No significant decrease in the activities of AChE and CES was observed in the cells treated with TCPP and TNPP for 24 h compared to the control group (Figure 4A,B). These results suggest that the two OPs did not affect the activities of the two phosphatase enzymes at the concentrations tested.

### 3.5. CEBPA Is One of the CYTOR Transcription Factors

**Figure 5:**
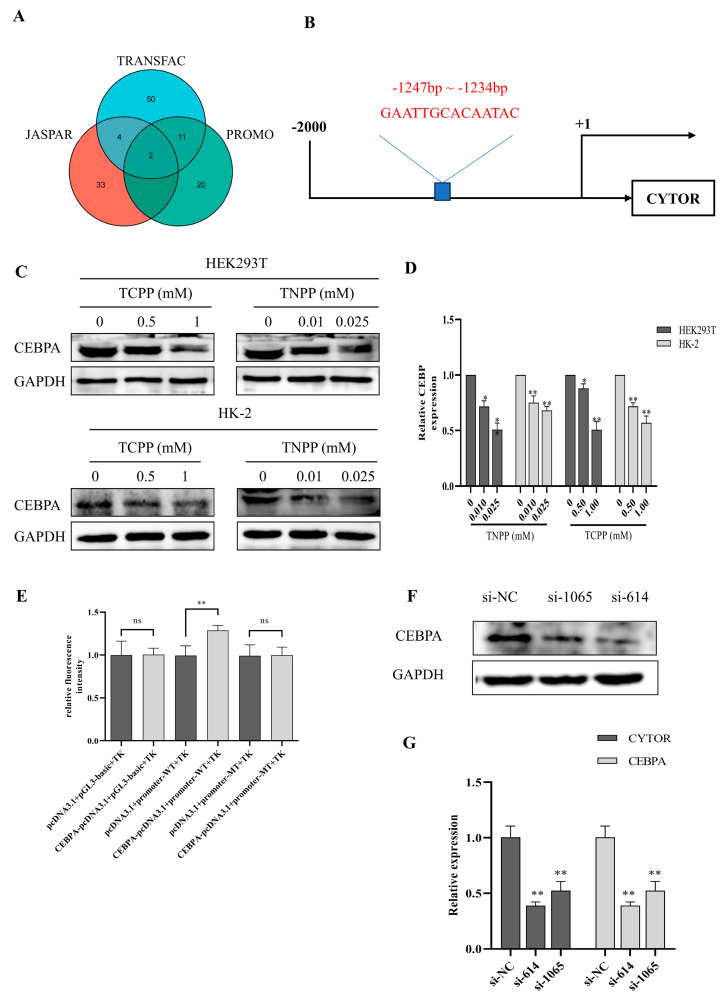
TCPP and TNPP downregulate CYTOR expression by reducing CEBPA levels. (**A**) The intersection number of CYTOR transcription factors predicted using the three databases-PROMO, JASPAR, and Alibaba2.0. (**B**) Binding sites of transcription factor CEBPA with CYTOR. (**C**) Western blot analysis of CEBPA in HEK293T and HK-2 cells after treatment with TCPP and TNPP. (**D**) Quantization of protein levels from Western blot analysis. Data are presented as the mean ± SD (* *p* < 0.05, ** *p* < 0.01, *n* = 3). (**E**) Relative fluorescence intensity detected after the co-transfection of each group of plasmids. Data are presented as the mean ± SD (** *p* < 0.01, ns indicates not significant, *n* = 3). (**F**) Western blot analysis of CEBPA protein expression in HEK293T cells after siRNA si-614 and si-1065 interference. (**G**) Expression level of CYTOR in HEK293T cells after CEBPA interference by siRNA. Data are presented as the mean ± SD (** *p* < 0.01, *n* = 3).

We predicted and validated the transcription factors of CYTOR to find out whether OPs lead to the downregulation of CYTOR expression through these factors. Using the prediction results from three transcription factor databases—PROMO, JASPAR, and Alibaba 2.0—we found that the transcription factor CEBPA could bind to positions 753–766 in the upstream region of the CYTOR promoter (Figure 5A,B). Western blot results showed that after treatment with TCPP and TNPP, CEBPA protein levels in HEK293T and HK-2 cells were decreased to different degrees, which was consistent with the downregulation of CYTOR expression (Figure 5C,D). Further, the dual luciferase assay showed that the relative fluorescence intensity measured in CEBPA-pcDNA3.1 and promoter-WT co-transfected cells was significantly higher than that of the other transfected groups, suggesting that the transcription factor can structurally bind to this site (Figure 5E). When the expression of CEBPA was knocked down with siRNA (si-614 and si-1065), CYTOR expression also decreased significantly (Figure 5F,G). These results indicate that the transcription factor CEBPA has the function of promoting the expression of CYTOR, while TCPP and TNPP can downregulate CYTOR expression by decreasing the levels of CEBPA in renal cells.

### 3.6. TCPP and TNPP Inhabited the Phosphorylation of ERK1/2

**Figure 6:**
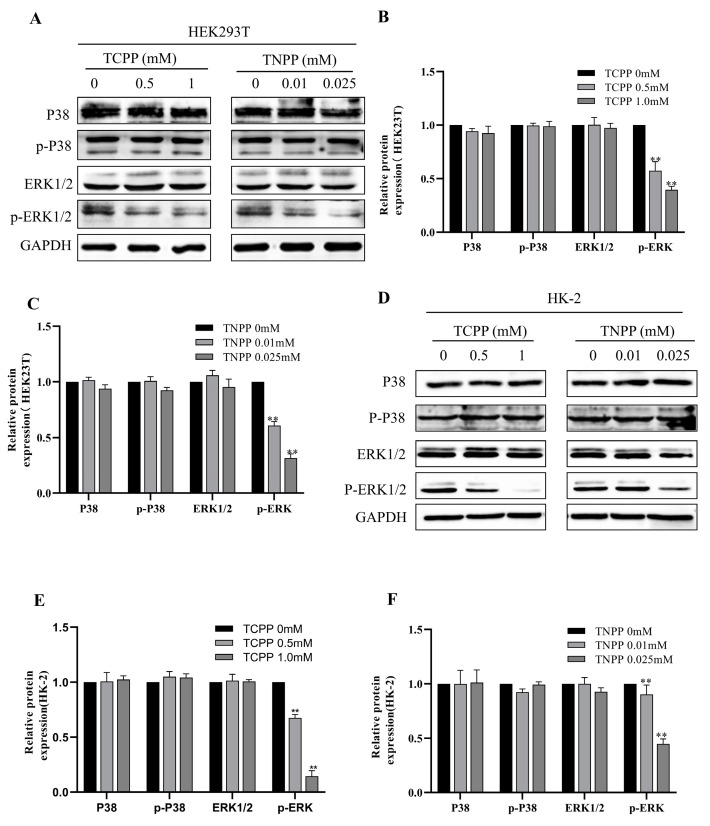
TCPP and TNPP downregulate CEBPA expression through inhibiting the phosphorylation of ERK1/2. (**A**) Western blot analysis of p38, p-p38, ERK1/2, and p-ERK1/2 protein expression in HEK293T after treatment with TCPP and TNPP. (**B**) Quantization of protein levels from Western blot analysis in HEK293T after TCPP treatment. Data are presented as the mean ± SD (** *p* < 0.01, *n* = 3). (**C**) Quantization of protein levels from Western blot analysis in HEK293T after TNPP treatment. Data are presented as the mean ± SD (** *p* < 0.01, *n* = 3). (**D**) Western blot analysis of p38, p-p38, ERK1/2, and p-ERK1/2 protein expression in HK-2 after treatment with TCPP and TNPP. (**E**) Quantization of protein levels from Western blot analysis in HK-2 after TCPP treatment. Data are presented as the mean ± SD (** *p* < 0.01, *n* = 3). (**F**) Quantization of protein levels from Western blot analysis in HK-2 after TNPP treatment. Data are presented as the mean ± SD (** *p* < 0.01, *n* = 3).

The MAPK signaling pathway is an important signaling pathway responsible for cellular proliferation [20]. We examined the expression and phosphorylation levels of two important upstream factors in this pathway, namely ERK and P38. The results showed that the protein levels of P38, p-P38, and ERK1/2 were not significantly changed in both renal cells after treatment with TCPP and TNPP, while the protein expression of p-ERK1/2 was decreased (Figure 6A–F). Since CEBPA is a downstream protein of ERK, we suggest that TCPP and TNPP can downregulate the expression of CYTOR via ERK/CEBPA, thereby increasing the induction of apoptosis and inhibiting the growth of renal cell lines.

## 4. Discussion

The cytotoxicity of OPs is mainly characterized by the inhibition of cell proliferation and the induction of apoptosis [21]. Long non-coding RNAs are widely involved in physiological processes such as cellular stress response, proliferation, and apoptosis [22,23]. However, the specific role of CYTOR in OP-induced cytotoxicity remains unclear. This study demonstrates that TCPP and TNPP, two commonly used industrial organophosphorus compounds, reduce renal cell survival by inducing apoptosis over a range of concentrations, doing so by downregulating the expression of lncRNA CYTOR through the ERK-CEBPA signaling pathway, a process that does not involve the inhibition of acetylcholinesterase and carboxylesterase activities. The results of this study provide a new perspective on the mechanism of organophosphorus toxicity.

TCPP and TNPP are mainly used in building materials and food packaging, respectively [24,25]. In the present study, we found that TCPP and TNPP exposure led to a decreased viability, an increase in ROS, and the induction of apoptosis in human kidney cells, according to the CCK-8 and flow cytometric assays. These results were consistent with some experimental results [26] that indicated that renal cell viability was negatively correlated with the concentration of TCPP. One study found that low concentrations of TCPP had pro-apoptotic and pro-activating effects on venous endothelial cells [27], but another study did not detect a significant increase in the rate of apoptosis and ROS in TCPP-treated hepatocytes [28], suggesting that the toxic effects of OPs are somewhat tissue/cell specific. ROS production is usually caused by a change in mitochondrial permeability [29]. We hypothesize that TCPP and TNPP, as lipophilic molecules, disrupt the mitochondrial cellular lipid membrane structure and exacerbate ROS accumulation. The IC_50_ values showed that TNPP with aryl groups had a greater inhibitory effect than TCPP in both renal cell types in our study. Similarly, one study found that aryl-containing TPP treated hepatocytes with a more pronounced inhibitory effect than TCPP [30], suggesting that aryl-containing OPs have a higher cytotoxicity. This may be due to the electron-capturing effect of the aryl group, which decreases the density of the molecular electron cloud and makes the molecule have an overall positive charge [31], so we speculated that the positively charged TNPP could combine with the biological anion easier, leading to an increased toxicity.

The inhibition of esterase activities such as CES and AChE in cells is one of the important ways in which organophosphorus compounds exert cytotoxicity [32]. In the present study, we found that both AChE and CES activities were not inhibited in renal cells after exposure to TCPP and TNPP. It was found that most alkyl OPs including TCPP did not possess the ability to inhibit CES in rat livers [33]. In addition, a study in a rat model showed that TDCPP and TCEP did not inhibit AChE in the brain and serum, but mainly caused neurotoxicity through non-cholinergic mechanisms [34]. Based on these results, the inability of TCPP and TNPP to inhibit AChE and CES may be due to their chemical structure, which also implies that these OPs induce cytotoxicity through non-cholinergic mechanisms.

CYTOR as a non-coding RNA usually plays the role of an oncogene in cancer, but it can also regulate cell growth in normal cells. One study found that the overexpression of CYTOR in cardiomyocytes inhibited apoptotic proteins Caspase-3 and Bax [35], thus reducing the apoptosis rate; they also found that CYTOR could upregulate Superoxide dismutase (SOD) and inhibit Malondialdehyde (MDA) expression to reduce oxidative stress in cells. In this study, TCPP and TNPP could downregulate the expression of CYTOR in kidney cells, accompanied by an increase in the levels of ROS and the promotion of apoptosis, while the overexpression of CYTOR could reduce the rate of apoptosis. It is suggested that lncRNA also plays a role in regulating apoptosis during the toxicogenic process of exogenous compounds.

Some studies have been reported on transcription factors that promote pathological processes by regulating CYTOR. The transcription factor SP1 promotes the expression of CYTOR, which, in turn, promotes the metastasis of cancer cells through the PI3K/AKT pathway [36]. The transcription factor Yes-associated protein 1 (YAP1) can interact with another transcription factor, transcriptional enhanced associate domain (TEAD), to indirectly regulate CYTOR, thereby promoting rectal carcinogenesis [37]. In triple-negative breast cancer, the transcription factor YIN-YANG 1 (YY1) was found to inhibit the transcription of CYTOR, thereby acting as an anticancer agent [38]. In this study, we confirmed that the transcription factor CEBPA binds to the promoter region of CYTOR and is a facilitator transcription factor for CYTOR during TCPP and TNPP toxigenicity. CEBPA performs transcriptional activation mainly through the C-terminal dimer formation and DNA binding, as well as through the N-terminal [39].

The mitogen-activated protein kinase (MAPK) signaling pathway plays an important role in the regulation of cell proliferation, in which subfamily protein ERK is a class of widely expressed protein kinases and intracellular signaling molecules that can respond to external stimuli to the cell; the phosphorylation of ERK generates kinase activity and activates many transcription factors, as well as some downstream protein kinases involved in the regulation of cell proliferation [40]. In the present study, the level of p-ERK1/2 in the MAPK signaling pathway was significantly downregulated after treatment with TCPP and TNPP, a result that is partially consistent with the results of the experiments in HepG2 cells [30], suggesting that the two OPs can inhibit renal cell proliferation through the MAPK signaling pathway. Studies have shown that the level of the CEBP protein family is regulated by p-ERK phosphorylation in a variety of cells; for example, activated p-ERK stimulates CEBPB expression in interferon-treated RAW cells [41], the activation of the p-ERK protein in 3T3-L1 cells promotes the expression of CEBPA, which in turn promotes adipogenesis [42]. In this study, the total ERK1/2 protein was not significantly changed in the OP-treated cell group compared to the control group, while both p-ERK1/2 and CEBPA were inhibited. Therefore, it was demonstrated that OPs could inhibit the activation of ERK, leading to a decrease in the expression of CEBPA, which in turn inhibited the transcriptional activity of CYTOR.

## 5. Conclusions

In summary, TCPP and TNPP inhibit ERK phosphorylation in the MAPK signaling pathway, leading to the downregulation of CEBPA expression. This, in turn, inhibits the transcriptional activity of CYTOR and weakens its anti-apoptotic function, leading to a reduced renal cell viability. Studies have shown that the ERK/CEBPA/CYTOR axis plays an important role in the regulation of organophosphorus-induced apoptosis in renal cells.

## Data Availability

The data that support the findings of this study are available from the corresponding author upon reasonable request.
